# Body image in patients with different types of cancer

**DOI:** 10.1371/journal.pone.0260602

**Published:** 2021-11-29

**Authors:** Jan Brederecke, Anja Heise, Tanja Zimmermann

**Affiliations:** Department of Psychosomatic Medicine and Psychotherapy, Hannover Medical School, Hannover, Germany; Rutgers Cancer Institute of New Jersey, UNITED STATES

## Abstract

**Background:**

Cancer can cause physical changes and affect satisfaction with a persons’ physical appearance, which in turn can affect overall quality of life. Previous studies have primarily focused on women with breast cancer and few is known about body image in patients with other cancers and especially men. The present study compares satisfaction with body image of patients with different types of cancer with the general population and across sexes and identifies risk factors for diminished body image. Additionally, patients that were diagnosed within the last year and those living with cancer for longer are compared.

**Methods:**

In this cross-sectional study, *N* = 531 cancer patients answered the German Self-Image Scale to assess body image. One sample *t*-tests are utilized to compare the body image of cancer patients with the general population. Stepwise regression analyses were used to identify factors associated with body image and ANOVAs with posthoc tests as well as *t*-tests were used to examine group differences.

**Results:**

Cancer patients showed diminished body image compared to the general population. For men, higher relationship satisfaction and lower cancer-specific distress were associated with more positive body self-acceptance (SA), whereas younger age, higher relationship satisfaction, and lower cancer-specific distress resulted in better perceived partner-acceptance of one’s body (PA). In women, higher education, lower anxiety and cancer-specific distress were associated with more positive SA. Female cancer patients with breast/gynecological cancer reported better SA than those with visceral cancers. Higher relationship satisfaction and lower cancer-specific distress were found to be associated with more satisfactory PA in females. Time since diagnosis did not affect body image in this study.

**Conclusions:**

Results indicate that cancer patients regardless of sex tend to have decreased body image satisfaction. Future research directions include examination of additional entities of cancer, deeper research in men and the role of time since diagnosis.

## Introduction

With approximately 19.3 million new cancer diagnoses and an estimated 9.9 million deaths from cancer worldwide in 2020, cancer is currently one of the leading global health concerns [1]. At the same time, modern cancer treatments have led to an increased life expectancy of those affected, and the number of people living with and surviving cancer is growing as well [2]. In Germany, more than 1.7 million people have been diagnosed with cancer within the last five years [3]. In the long term, many patients experience not only physical complaints but also psychosocial problems such as anxiety and depression [4], sleep disturbances [5], post-traumatic growth [6], the impact of cancer on relationships [7], and fear of cancer recurrence [8]. Additionally, the survivor’s body image has long been of researchers’ interest due to the body-altering effect of cancer and many treatments. The body image construct in the domain of psycho-oncology can generally be conceptualized as multidimensional, including thoughts, feelings, and perceptions towards the own body, sexuality, and functionality and thus affecting a person’s quality of life [9–11].

Most of the body image-related research in the field of psycho-oncology focuses on the consequences of irreversible body alterations through surgical treatment (e.g. scars and amputations). Thus, researchers have assessed breast cancer surgery and its side effects [12], the impact of head and neck cancer treatment [13, 14], differences between colorectal cancer patients with and without a stoma [15], consequences of anal cancer treatment [16] and many more common scenarios. Also, the side effects of nonoperative therapies (e.g. chemotherapy, radiotherapy, hormone therapy) have been investigated intensively: hair loss [17], lymphedema [18], skin irritation and pain [19], and weight gain [20] amongst other side effects have been examined. As a result of all this, there is no doubt that cancer and its treatment can negatively affect a person’s body image. Furthermore, research indicates that a diminished body image satisfaction can have negative consequences on physical and psychological health, interpersonal relationships, lead to psychological distress and thus impair quality of life [21–26].

Most studies that investigate body image in cancer patients and survivors are exclusively conducted with female participants and focus nearly entirely on the most common cancer entities like breast cancer [10]. Studies with men and less frequent types of cancer are thus rare. Additionally, it is difficult to infer the actual extent to which cancer patients’ body image is impaired when compared to the general population as the number of case-control studies is also very limited [27]. In their 2015 systematic review, Lehmann, Hagedoorn, and Tuinman reported 25 studies that compared cancer survivors with control groups from the general population [27]. The authors could not find clear evidence that cancer survivors report decreased satisfaction with their bodies when compared to the general population as nearly 50% of the studies did not report differences between healthy and cancer populations, and some studies even found cancer survivors to have a more positive body image [27]. The ambiguous results were additionally amplified by the heterogeneity in measures and the specific aspects of body image that were assessed [27]. Furthermore, several of the measures were experimenter derived and some studies had other relevant methodological issues [27]. As most studies regarding body image in cancer patients and survivors are conducted in female breast cancer patients, many of the common measures were especially developed and validated for this population only, making comparisons to other cancer types or the general population difficult [28].

Another area that has hardly been explored so far in psycho-oncological body image research is intimate relationships. Nonetheless, close relationships have been shown to be important sources of coping with cancer [29] and spousal reactions, as well as marital satisfaction, are thus likely to influence body image difficulties [10]. The general literature on body image and intimate relationships reports that the social dimension of the bodily self-image can be influenced by intimate relationships, especially for women but also for men [30]. This assumption is supported by studies showing, that body image satisfaction and sexual satisfaction are equally associated with perceived relationship quality in men as well as women [31]. In women diagnosed with breast cancer who are married or in committed relationships, a conjoint process of mutual support was found to even be an effective coping strategy as well [32]. This dyadic consideration also raises the question of a dyadic study of body image. Previous studies have examined body image from the patient’s perspective. However, there is a lack of studies that also examine the perception of body image from the partners’ perspective. The present study aims to achieve this by examining body image from two perspectives: body self-acceptance (SA; patient’s perspective) and perceived partner acceptance of one’s body (PA; partner’s perspective).

### Study objectives

The present study aimed to gain more information on cancer patients’ body image in general. Therefore, the *first goal* was to add to the currently still ambivalent literature on body image comparisons between cancer patients and the general population. Thus, the German version of the Self Image Scale (SIS-D) was utilized as a measure for body image satisfaction as it provides normative scale scores for the German general population [33] and was validated in a diverse sample of cancer patients in previous studies [34]. Moreover, the SIS-D incorporates the relational component of body image, the perceived partner perspective on a person’s body via the *partner-acceptance* scale.

The *second goal* was to determine factors associated with the body image satisfaction of cancer patients across cancer entities. While only few studies examined predictors for body image in cancer patients, they mostly focus on medical variables like the type of treatment and were mostly restricted to female breast cancer patients (e.g. [35, 36]). The present study focuses on demographic variables like age, sex, and educational status as well as anxiety, depression, cancer-specific distress, and relationship satisfaction in a sample with different cancer entities. These variables have also been studied before but again predominantly in female breast cancer patients or the general population. The available research shows younger age to be a possible predictor of female breast cancer patients’ diminished body image satisfaction [36, 37] while its role in other groups of cancer patients remains unclear and the tendency seems to be the reverse in the general population [33]. In contrast, the role of sex differences can be expected to be more straight forward as the available research on body image in the general population strongly indicates that women consistently report lower body image satisfaction than men [33, 38, 39]. Educational levels have also been linked to body image satisfaction in female breast cancer patients [40, 41] in that higher education might predict less positive body image. Its role in cancer patients body image satisfaction in general remains unclear, though. A predictive value of depression has also been reported previously (e.g. [42]) as it is strongly associated with body image difficulties in the general population [43] and female breast cancer patients (e.g. [42]) as depressed people tend to see their body more negatively. A similar relationship with body image can generally be expected from the reported levels of anxiety due to the strong overlap and comorbidity with depression as well as indicating study results in the general population [33]. Another related construct that has been linked to the prediction of decreased body image satisfaction in female breast cancer survivors is cancer-specific distress (e.g. [44]). Additionally, increased relationship satisfaction of the patient has been shown to predict more satisfactory levels in the partner-acceptance facet of female breast cancer patients [42].

Moreover, the *third goal* was to examine body image differences in cancer entities. While the idea that different types of cancer differ regarding their body image satisfaction is not new and differences between cancer types have repeatedly been examined, e.g. between men with prostate or laryngeal cancer [45] or in patients with head and neck cancer [13], a general quantification of the differences is not yet widely possible through the available literature. The present study aims to contribute to the scarce literature available to date by comparing patients with different entities of cancer.

*Finally*, part of the available literature which again mainly focusses on female breast cancer patients indicates that cancer patients’ body image satisfaction improves over time (e.g. [46, 47]). At the same time contradictory findings exist as well, indicating a more stable body image disturbance after breast cancer [44]. The unclear findings and missing data on the role of the time since diagnosis in other cancer patients are the basis for the last research question of the present study. Differences between patients that were more recently diagnosed with cancer and those that have been living with cancer for a longer time were to be compared. In order to achieve reasonable groupsizes for comparison, the categories of diagnosed in the last year (<1 year) and diagnosed more than a year ago (>1 year) were chosen. In addition, it can be assumed that after one year certain physical changes have already healed (such as scars or hair loss).

## Methods

### Data sampling

Cancer patients of full age with German language skills and without recognizable mental disorders were included (inclusion criteria). The participants were recruited in both inpatient (rehabilitation clinic Oberharz (April to September 2016), municipal hospital Braunschweig (February 2006 to February 2009), and university hospital Munich (February 2006 to June 2007)) and outpatient settings (oncology practices in Braunschweig (February 2002 to October 2013)). Patients were informed about the study at the clinics or practices by information material. In case of interest, contact was made by the study team. In accordance with agreement and consent, the participants were asked to fill out paper-pencil versions of the survey. Moreover, all participants provided basic demographic information and accepted written informed consent before they participated in the study. The study has been approved by the ethics committees of Technical University Braunschweig (B-2016-05), German Society of Psychology (TZ10.2009), and Hanover Medical School (2106–2016).

Of the *N* = 536 cancer patients that originally took part in the survey, *n* = 5 (93%) were excluded as one male patient with breast cancer could not be meaningfully examined and four patients did not report their sex which was deemed necessary for the imputation process of missing values. Thus, the final sample size resulted in *N* = 531 participants.

### Measures

#### Body image satisfaction

The Self-Image Scale (SIS) was originally designed to measure body image adjustment in women with cancer, especially breast and gynecological cancer. Zimmermann, Scott, and Heinrichs [42] did the original translation of the SIS into German. The SIS-D was validated in a representative sample of the general German population [33]. For the study, all femininity specific items were presented in a masculinity specific version as well (e.g., “I think my partner sees me as a woman/man”) to include men. The SIS-D showed good psychometric properties and is thus fit for use in men as well as women. The SIS-D consists of eleven items that are assigned to two scales: *self-acceptance* (six items) that assesses sense of femininity/masculinity as well as acceptance of appearance (e.g., “I like the way I look undressed”) and *partner-acceptance* (five items) that assesses participants’ perceptions of their partner’s responses to, and acceptance of their appearance (e.g., “I think my partner finds me attractive”). The SIS-D asks for this evaluation regarding the past week. Items are rated on a 5-point Likert-scale ranging from 1 (*strongly disagree*) to 5 (*strongly agree*). Two items are negatively formulated and thus have to be recoded afterward. The self-acceptance scale score ranges from 6 to 30 while the partner-acceptance scale score ranges from 5 to 25. Higher scores suggest greater acceptance. The validation study suggested good psychometric qualities with internal consistencies (Cronbach’s α; [48]) of .82 for self-acceptance and .89 for partner-acceptance [33]. Cronbach’s α in the present study was .81 for self-acceptance and .87 for partner-acceptance. In the present study, the SIS-D’s self-acceptance scale was used as the measure for SA while the partner-acceptance scale was used as the measure for PA.

#### Depression and anxiety

The German version of the Hospital Anxiety and Depression Scale (HADS-D) [49] assesses symptoms of anxiety and depression using 14 items, seven of which relate to symptoms of anxiety and seven relate to depressive symptoms. Both scales use a cut-off score of 8 to indicate the presence of either a depressive or an anxiety disorder. The internal consistencies of the HADS-D subscales were α = .80 for both scales [49]. Internal consistencies in the present study were α = .83 for the anxiety-scale and α = .84 for the depression scale. Scale scores for the PHQ-9, GAD-7, and HADS-D were standardized to allow for meaningful comparisons across the subsets regarding depression and anxiety.

Depression was also assessed using the German version of the PHQ-9 [50] in some subsets. Items are rated on a 4-point Likert-type- scale ranging from 0 (*not at all*) to 3 (*nearly every day*). The PHQ-9 total score ranges from 0 to 27. In the original study, the PHQ-9 showed excellent internal consistency with α ranging from .86 to .89 [50]. In this study, the internal consistency was α = .85.

Anxiety was also assessed using the German version of the General Anxiety Disorder Questionnaire (GAD-7; [51]) in some of the subsets. Items are rated on a 4-point Likert-type- scale ranging from 0 (*not at all*) to 3 (*nearly every day*). The GAD-7 total score ranges from 0 to 21. In the original study, the GAD-7 showed good internal consistency with α = .92 [51]. In this study, the internal consistency was α = .87.

#### Relationship satisfaction

The German version of the Quality of Marriage Index (QMI-D; [52, 53]) assesses relationship quality. The self-evaluation tool includes six items of which five are answered on a 7-point Likert-type scale (1: *strongly disagree*; 7: *strongly agree*) and one is answered on a 10-point Likert-type scale (1: *extremely low*; 10: *extremely high*). The total sum score ranges from 6 to 45 with higher scores indicating better relationship quality. Cut off values below 34 indicate low partnership quality. The QMI-D’s internal consistency is excellent with α = .94 [52] and Cronbach’s alpha in the current sample with α = .96.

#### Cancer-specific distress

Cancer-specific concerns and distress were assessed with the German version of the Questionnaire on Stress in Cancer Patients (FBK-R23 [54]). The FBK-R23 provides 23 cancer-specific stress situations. First, the patient replies to the question of whether the given problem is currently occurring and, if so, to what extent it causes distress. The response categories range from 0 (*the problem does not apply to me*) to 5 (*the problem applies to me and is a very big problem*). The FBK-R23’s α for the total score was .92 in the present study which is equally good as in the original study [α = .89; 54]. Total scores range from 0 to 115 and the cut-off for clinical distress related to cancer is 34.

#### Fear of cancer recurrence

The German version of the Fear of Progression Questionnaire Short Form (PA-F-KF [55]) measures the fear of cancer recurrence. The 12 items are answered on a 5-point Likert type scale ranging from 1 (*never*) to 5 (*very often*). All item scores are added up to a sum score with higher values indicating higher levels of fear of progression (range: 5–60). A cut-off of 34 indicates a dysfunctional fear of progression. The PA-F-KF had an α of .91 in the present study and an α of .87 in the validation study [55].

### Statistical analysis

Bivariate associations between metric variables were calculated using Pearson-correlations (*r*), associations between categorical variables were calculated using χ^2^-tests, bivariate associations between metric and ordinal variables were calculated using Spearman’s rank correlation (ρ), and associations of nominal and metric variables were calculated using the eta coefficient (η). Bidirectional stepwise linear regression analyses were used to determine factors associated with the different facets of body image in cancer patients. Categorical variables with more than two categories were dummy coded and education was treated as an ordered factor. Group differences were calculated using *t*-tests or ANOVAs with Tukey’s honest significance test (Tukey HSD; [56]) as a posthoc test. The statistical analyses were performed in *R* 4.0.3 [57]. All tests were based on a significance level of .05 (if not stated otherwise). Missing values (~5%) were imputed using *m* = 20 multiple imputations that were created via the *mice* package [58] as missing values were assumed to be at least missing at random. Thus all calculations but the stepwise regressions were done with the 20 completed datasets and results were pooled according to *Rubin’s rules* where necessary [59]. The stepwise regressions were done using one randomly selected complete dataset from the pool of complete datasets as the variable reducing nature of the procedure would have introduced errors otherwise.

## Results

### Sample characteristics

Participants were diagnosed with a range of different cancer types as shown in Table 1. The sample (*N* = 531) consisted of 286 (53.86%) women and 245 (46.14%) men with a mean age of 61.91 years for men (*SD* = 12.12, range:23–83) and 54.93 years for women (*SD* = 9.87, range:27–81). Men and women differed significantly in all demographic variables except for the amount of those living in a relationship. Men and women also differed regarding all of the used questionnaires (see Table 1). The majority of the participants (90.21%; *n* = 479) was currently living in a relationship and was thus suitable for further investigation regarding the perceived partner-acceptance aspect of body image as well as all analyses that included relationship quality. To obtain reasonable group sizes, cancer entities were grouped into four groups: (1) breast and gynecological cancers, (2) prostate and testicular cancers, (3) visceral cancers, and (4) others as has been done in prior studies (e.g. [34]). Visceral cancers include lung, colorectal, and stomach cancer while the category others included skin cancer, lymph node cancer, leukemia, and others. Men and women additionally differed significantly regarding the time since diagnosis (*t*(340.31) = -4.815, *p* < .001) with *M* = 26.72 (*SD* = 32.81) months for men and *M =* 15.07 (*SD* = 20.32) months for women.

**Table 1:** Demographic characteristics of the total sample (*N* = 531) and by sex.

	Total (*N* = 531)	Women (*n* = 286)	Men (*n* = 245)	*p* ^a^
**Age in years** (*M*, *SD*)	58.15 (11.50)	54.93 (9.87)	61.91 (12.12)	**< .001** ^b^
**Years of education** (*n*, %)				**.020** ^c^
≤9	243 (45.73%)	113 (39.44%)	130 (53.06%)	
10	125 (23.58%)	75 (26.29%)	50 (20.41%)	
>10	150 (28.25%)	90 (31.47%)	60 (24.49%)	
Other	13 (2.45%)	8 (2.80%)	5 (2.04%)	
**Type of cancer** (*n*, %)				**< .001** ^c^
Breast & gynecological	222 (41.85%)	222 (77.71%)	-	
Prostate & testicular	110 (20.78%)	-	110 (45.04%)	
Visceral	70 (13.12%)	27 (9.51%)	42 (17.33%)	
Other	129 (24.25%)	37 (12.78%)	92 (37.63%)	
**Time since diagnosis**				
>1 year (*n*, %)	228 (42.85)	107 (37.57)	120 (49.02)	**.012** ^c^
In months (*M*, *SD*)	20.45 (26.76)	15.07 (19.37)	26.72 (32.31)	**< .001** ^b^
**Relationship**				
In a relationship (*n*, %)	479 (90.21%)	252 (88.11%)	227 (92.65%)	.110^c^
Duration in years (*M*, *SD*)	30.50 (15.46)	26.58 (14.56)	34.85 (15.29)	**< .001** ^b^
**Depression** (*M*, *SD*)^d^	0 (1)	.19 (1.04)	-.22 (.91)	**< .001** ^b^
**Anxiety** (*M*, *SD*)^d^	0 (1)	.17 (1.04)	-.20 (.92)	**< .001** ^b^
**Self-acceptance** (*M*, *SD*)^e^	20.85 (4.26)	20.31 (4.40)	21.49 (4.01)	**.001** ^b^
**Partner-acceptance** (*M*, *SD*)^e^	18.17 (4.18)	18.57 (4.06)	17.72 (4.28)	**.026** ^b^
**Relationship satisfaction** (*M*, *SD*)^f^	37.88 (7.76)	37.05 (8.58)	38.79 (6.62)	**.014** ^b^
**Fear of cancer recurrence** (*M*, *SD*)^g^	31.64 (10.47)	34.73 (9.84)	28.04 (10.05)	**< .001** ^b^
**Cancer-specific distress** (*M*, *SD*)^h^	31.90 (21.41)	36.01 (22.17)	27.11 (19.45)	**< .001** ^b^

Notes.

^a^*p* of the sex comparison.

^b^*t*-test.

^c^χ^2^-test.

^d^z-standardized scale scores of either the German Patient Health Questionnaire (PHQ-9) and General Anxiety Disorder Questionnaire (GAD-7) or the German Hospital Anxiety and Depression Scale (HADS-D).

^e^measured with the German version of the Self-Image Scale (SIS-D).

^f^measured with the German Quality of Marriage Index (QMI-D).

^g^measured with the German version of the Fear of Progression Questionnaire.

^h^measured with the German version of the Questionnaire on Stress in Cancer Patients (FBK-R23).

### Body image satisfaction of cancer patients compared to the general population

The SIS-D’s self-acceptance and partner-acceptance scores were compared to the normative subscale scores available for the SIS-D. Norms for different age groups and males and females are available [33]. Male cancer patients in the present sample differed significantly from the general population regarding self-acceptance scores with a mean difference (*M*Δ) of -1.28 (*t*(239.27) = -4.963, *p* < .001) and partner-acceptance scores (*t*(218.42) = -6.744, *p* < .001, *M*Δ = -1.93). The same applied to female cancer patients with regard to self-acceptance (*t*(272.58) = -6.798, *p* < .001, *M*Δ = -1.79) as well as partner-acceptance (*t*(240.69) = -4.741, *p* < .001, *M*Δ = -1.23). Thus, both groups reported less satisfactory SA and PA when compared to the general population.

### Factors associated with body image satisfaction in cancer patients

As men and women differed significantly across the self-acceptance and partner-acceptance facets of body image (see Table 1), it was decided to further examine them separately. Stepwise linear regression models for each sex were used to examine the SA and PA facets of body image and thus prevent problems that could arise from multicollinearity as constructs like depression, anxiety, and cancer-specific distress tend to be highly correlated. To include a reasonable number of factors in the models, bivariate correlations were used to determine and include only those variables that were significantly related to the SIS-D’s self-acceptance and partner-acceptance scales (see Table 2).

**Table 2:** Correlations with self-acceptance and partner-acceptance in cancer patients by sex (*N* = 531).

	Men (*n* = 245)		Women (*n* = 286)	
	Self-acceptance	Partner-acceptance	Self-acceptance	Partner-acceptance
	(*n* = 245)	(*n* = 227)	(*n* = 286)	(*n* = 252)
Age (*p*)	-.09 (.156)	**-.18 (.004)**	-.08 (.207)	-.10 (.109)
Education (*p*)^a^^,^^b^	.09 (.163)	.00 (.954)	**.23 (.000)**	.03 (.575)
Cancer entity (*p*)^c^	.05 (.791)	.15 (.085)	**.18 (.010)**	.14 (.100)
Time since diagnosis (*p*)	-.06 (.336)	-.09 (.155)	-.07 (.261)	-.10 (.100)
Duration of relationship (*p*)	-.11 (.088)	**-.17 (.010)**	-.05 (.431)	-.01 (.936)
Depression (*p*)^d^	**-.39 (.000)**	**-.30 (.000)**	**-.39 (.000)**	**-.22 (.000)**
Anxiety (*p*)^d^	**-.40 (.000)**	**-.22 (.000)**	**-.42 (.000)**	**-.21 (.001)**
Relationship satisfaction (*p*)^e^	**.39 (.000)**	**.33 (.000)**	**.15 (.010)**	**.46 (.000)**
Fear of cancer recurrence (*p*)^f^	**-.34 (.000)**	**-.23 (.001)**	**-.35 (.000)**	**-.22 (.000)**
Cancer-specific distress (*p*)^g^	**-.44 (.000)**	**-.32 (.000)**	**-.39 (.000)**	**-.33 (.000)**

Notes.

^a^Spearman rank correlation (ρ).

^b^The “other” category was excluded in order to provide meaningful categories for the rank correlations resulting in sample sizes of *n* = 240 for male self-acceptance, *n* = 222 for male partner-acceptance, *n* = 278 for female self-acceptance, and *n* = 244 for female partner-acceptance.

^c^Eta (η).

^d^z-standardized scale scores of either the German Patient Health Questionnaire (PHQ-9) and General Anxiety Disorder Questionnaire (GAD-7) or the German Hospital Anxiety and Depression Scale (HADS-D).

^e^measured with the German Quality of Marriage Index (QMI-D), sample sizes were analogous to the respective patients living in a relationship.

^f^measured with the German version of the Fear of Progression Questionnaire (PA-F-KF).

^g^measured with the German version of the Questionnaire on Stress in Cancer Patients (FBK-R23). Bold values indicate *p* < .05.

#### Factors associated with body image satisfaction in male cancer patients

As shown in Table 2, the self-acceptance scale in men covaried significantly with depression, anxiety, relationship satisfaction, fear of cancer recurrence, and cancer-specific distress. Thus, the mentioned variables were included in the respective stepwise regression analysis. This had the effect that only the 227 (92.65%) men who were living in a relationship were included in the regression analysis. A separate analysis of the remaining 18 men that were not living in a relationship was not considered statistically purposeful. The resulting model (*R*^2^ = .266, *F*(2, 224) = 41.86, *p* < .001) displayed in Table 3 showed higher relationship satisfaction and lower levels of cancer-specific distress scores to be statistically significant factors associated with higher self-acceptance scores and thus more positive SA in male cancer patients.

**Table 3:** Results of the stepwise regression analysis for self-acceptance in men (*n* = 227).

Effect	Estimate	*SE*	95% CI		*p*
			LL	UL	
Intercept	16.80	1.54	[13.80, 19.90]		< .001***
Relationship satisfaction^a^	.17	.04	[.10, .24]		< .001***
Cancer-specific distress^b^	-.07	.01	[-.10, -.05]		< .001***

Notes.

^a^measured with the German Quality of Marriage Index (QMI-D).

^b^measured with the German version of the Questionnaire on Stress in Cancer Patients (FBK-R23).

***p < .001.

**p < .01.

*p < .05.

Male partner-acceptance covaried in a statistically significant way with age, duration of relationship, depression, anxiety, relationship satisfaction, fear of cancer recurrence, and cancer-specific distress scores as is shown in Table 2. The respective regression model (*R*^2^ = .227, *F*(3, 223) = 23.09, *p* < .001) showed that younger age, higher relationship satisfaction, and less cancer-specific distress were associated with higher levels of partner-acceptance, i.e. better SA in male patients (see Table 4).

**Table 4:** Results of the stepwise regression analysis for partner-acceptance in men (*n* = 227).

Effect	Estimate	*SE*	95% CI		*p*
			LL	UL	
Intercept	18.27	2.15	[14.0, 22.5]		< .001***
Age	-.10	.02	[-0.14, -0.06]		< .001***
Relationship satisfaction^a^	.19	.04	[0.11, 0.27]		< .001***
Cancer-specific distress^b^	-.06	.01	[-0.09, -0.03]		< .001***

Notes.

^a^measured with the German Quality of Marriage Index (QMI-D).

^b^measured with the German version of the Questionnaire on Stress in Cancer Patients (FBK-R23).

***p < .001.

#### Factors associated with body image satisfaction in female cancer patients

Women’s self-acceptance scores covaried significantly with education, the type of cancer, depression, anxiety, relationship satisfaction, fear of cancer recurrence, and cancer-specific distress (see Table 2). Therefore, these variables were included in the regression model. Only the 252 (88.11%) female participants in a relationship were included in the model calculation as the relationship quality was also in the pool of factors. The “other” category of the education variable was coded as missing to allow for a meaningful direction of the factor levels which excluded another 8 participants from the model. This model (*R*^2^ = .232, *F*(6, 237) = 13.22, *p* < .001) showed that higher educational levels, lower anxiety-levels and lower cancer-specific distress scores were statistically significant factors associated with increased female self-acceptance levels, i.e. more positive SA (see Table 5). Additionally, those with visceral cancers showed lower levels of self-acceptance compared to those with breast and gynecological cancers and were thus less satisfied with their bodies when controlled for all the other included variables (see Table 5).

**Table 5:** Results of the stepwise regression analysis for self-acceptance in women (*n* = 244).

Effect	Estimate	*SE*	95% CI			*p*
				LL	UL	
Intercept	22.70	.62	[20.30, 23.00]			< .001***
Years of education^a^	1.68	.42	[-.41, 2.00]			< .001***
Anxiety^b^	-.92	.33	[-1.56, -.20]			.006**
Cancer-specific distress^c^	-.05	.02	[-.08, -.00]			.001**
Cancer entity: other^d^	-1.00	.81	[-2.60, .60]			.222
Cancer entity: visceral^d^	-2.35	.84	[-4.00, -.60]			.006**

Notes.

^a^The education variable has been included as an ordered factor, the displayed values represent the linear correlation with the dependent variable. A quadratic relationship was not statistically significant and thus not included in the results (*p* = .530).

^b^z-standardized scale scores of either the General Anxiety Disorder Questionnaire (GAD-7) or the German Hospital Anxiety and Depression Scale’s anxiety-scale (HADS-D).

^c^measured with the German version of the Questionnaire on Stress in Cancer Patients (FBK-R23).

^d^The reference category is breast and gynecological cancers.

***p < .001.

**p < .01.

Depression, anxiety, relationship satisfaction, fear of cancer recurrence, and cancer-specific distress scores correlated in a statistically significant way with women’s levels of partner-acceptance and were thus included in the respective regression model. In this model (*R*^2^ = .226, *F*(2, 209) = 30.440, *p* < .001) higher relationship satisfaction and lower levels of cancer-specific distress were shown to be significant factors associated with higher partner-acceptance scores, i.e. a more satisfactory PA (see Table 6).

**Table 6:** Results of the stepwise regression analysis for partner-acceptance in women (*n* = 244).

Effect	Estimate	*SE*	95% CI		*p*
			LL	UL	
Intercept	13.00	1.26	[10.5, 15.4]		< .001***
Relationship satisfaction^a^	.19	.03	[.13, .24]		< .001***
Cancer-specific distress^b^	-.04	.01	[-.06, -.01]		.002**

Notes.

^a^measured with the German Quality of Marriage Index (QMI-D).

^b^measured with the German version of the Questionnaire on Stress in Cancer Patients (FBK-R23).

***p < .001.

**p < .01.

### Body image satisfaction in different cancer entities by sex

#### Body image satisfaction in different cancer entities in men

An ANOVA that compared the three applicable groups of cancer entities regarding self-acceptance scores in men (groups: prostate and testicular cancers, visceral cancers, and others) revealed no statistically significant differences between these groups (*F*(2, 15354.12) = .235, *p* = .791) as could be expected from the results of the η coefficient in Table 2. The means of self-acceptance were 21.36 (*SD* = 3.75) in prostate and testicular cancer patients, 21.33 (*SD* = 5.05) in visceral cancer patients, and 21.73 (*SD* = 3.80) in the group with other cancer entities.

As was also expected from the former correlations, no significant differences between the cancer entities regarding partner-acceptance scores in men emerged (*F*(2, 4466.123) = 2.465, *p* = .085). The means were 17.07 (*SD* = 4.34) in prostate and testicular cancer, 18.87 (*SD* = 4.48) in visceral cancer, and 18.02 (*SD* = 4.01) in patients with other cancer entities.

#### Body image satisfaction in different cancer entities in women

In the different groups of female cancer patients (breast and gynecological cancers, visceral cancers, and others), a statistically significant difference regarding self-acceptance scores emerged (*F*(2, 54866.738) = 4.629, *p* = .010) as could be inferred from the statistically significant η coefficient in Table 2. The means in self-acceptance scores were 20.68 (*SD* = 4.19) in breast and gynecological cancer patients, 18.10 (*SD* = 5.25) in visceral cancer patients, and 19.66 (*SD* = 4.53) in the group with other cancer entities. A Tukey HSD posthoc test revealed a significant difference (*p* = .004) between breast and gynecological cancer patients and visceral cancer patients.

The means of 18.85 (*SD* = 3.69) in breast and gynecological cancer patients, 17.98 (*SD* = 5.08) in visceral cancer patients, and 17.20 (*SD* = 5.25) in the group with other cancer entities that were observed in partner-acceptance scores of female cancer patients did not differ in a statistically significant way (*F*(2, 16239.023) = 2.302, *p* = .10).

### Differences in body image satisfaction in patients diagnosed less and more than a year ago

To determine whether patients that were diagnosed less than a year ago would show different levels of self- and partner-acceptance scores than those who already lived longer with cancer, *t*-tests were utilized. As men and women differed significantly in this categorical representation of the time since diagnosis, they were examined separately. Male patients living for a maximum of one year with their cancer diagnosis (self-acceptance: *M* = 21.96, *SD* = 4.06; partner-acceptance: *M* = 17.83, *SD* = 4.46) did neither differ regarding self-acceptance scores (*t*(209.96) = 1.409, *M*Δ = .75, *p* = .160) nor partner-acceptance scores (*t*(215.98) = .421, *M*Δ = .24, *p* = .674) from those who lived longer with their diagnosis (self-acceptance: *M* = 21.21, *SD* = 3.89, partner-acceptance: *M* = 17.59, *SD* = 4.19).

The same was found for female patients that were diagnosed within the last year (self-acceptance: *M* = 20.50, *SD* = 4.66, partner-acceptance: *M* = 18.63, *SD* = 4.16) when compared to those that were living for more than twelve months with cancer (self-acceptance: *M* = 19.92, *SD* = 4.46, partner-acceptance: *M* = 18.46, *SD* = 4.12). Thus, the women did neither differ in a statistically significant way regarding self-acceptance scores (*t*(153.81) = 0.928, *M*Δ = .57, *p* = .355) nor partner-acceptance scores (*t*(161.53) = 0.295, *M*Δ = .16, *p* = .769).

## Discussion

The present study examined the SA and PA facets of body image utilizing the self-acceptance and partner-acceptance scales of the SIS-D in a diverse sample of cancer patients. In contrast to the vast majority of previous studies regarding body image in cancer patients, the sample consisted of men, women, and a wide range of cancer diagnoses were included. Besides comparing the cancer patients’ self-acceptance and partner-acceptance scores to normative scale scores of the general population available for the SIS-D and comparisons of different cancer entities, possible factors associated with body image were examined and patients that were recently diagnosed were compared to patients that have been living with cancer for longer.

### Body image satisfaction of cancer patients compared to the general population

Cancer patients in the present study consistently showed decreased levels of both self-acceptance and partner-acceptance regardless of sex. Thus, men and women rated both their own acceptance as well as the perceived acceptance by their partners lower than the general population. This result is generally consistent with the majority of studies showing that a cancer diagnosis and its treatment negatively influence satisfaction with one’s body [22, 60] although opposing results exist as well [27]. Even though the differences between the sample and the normative scale scores of the SIS-D for the general population were statistically significant, the clinical relevance of the mean differences can not be inferred from the present study’s results.

### Factors associated with body image satisfaction

#### Factors associated with SA in men

Higher relationship satisfaction and lower levels of cancer-specific distress emerged as statistically significant factors associated with more satisfactory SA in males. These results regarding relationship satisfaction are in line with the broader body image literature that indicates that a feeling of closeness towards one’s spouse can improve a person’s view of themselves [61]. This can also be seen as in accordance with findings which suggest that satisfaction with partner support and functional dyadic coping lead to a better psychological adjustment to cancer [30, 62]. The association of patients’ cancer-specific distress with SA is also consistent with results in the literature on body image in cancer patients in general [63] as well as former findings in exclusively male samples of cancer patients [45].

#### Factors associated with PA in men

Statistically significant factors associated with more positive PA in men were young age, higher relationship satisfaction and lower levels of cancer-specific distress. The findings regarding age are consistent with the results of the SIS-D’s validation in the German general population [33]. It can generally be assumed that changes in health, appearance, and sexual functioning that come with aging can lead to the impression that partners might find one less attractive [64, 65]. This might partially explain the role age plays regarding men’s partner-acceptance as the SIS-D’s partner-acceptance scale focuses primarily on sexual attractiveness. Nonetheless, there are also hints that intimacy in the elderly is less driven by sexual activity and more by emotional intimacy [66]. The positive association of men’s reported PA and relationship satisfaction matches numerous studies’ results of satisfaction with partner support and dyadic coping increasing psychological adjustment to cancer (e.g. [30, 62]). Generally, men who are in a relationship when diagnosed with cancer are reported to cope better with the consequences of cancer and its treatment (e.g. [67]). The results on cancer-specific distress and PA are comparable to the results on SA and thus show that lower cancer-specific distress is associated with higher satisfaction with one’s body in general.

#### Factors associated with SA in women

In female participants, higher educational levels, lower levels of anxiety and cancer-specific distress as well as having breast or gynecological types of cancer emerged as significant factors associated with more positive SA. While the results of the present study might thus indicate a positive linear association of educational levels and women’s levels of self-acceptance, few is generally known about the impact of education on women’s SA and particularly female cancer patients’ SA. Nonetheless, a few studies suggest that higher education and income can predict more positive body image perceptions in female breast cancer patients [40, 41]. On the contrary, in the general population women with a higher socioeconomic status were found to be more dissatisfied with their bodies than socially disadvantaged women which in general have lower levels of education [68]. Consequently, the results regarding the predictive value of educational levels have to be interpreted very cautiously and future examinations should include this topic for further clarification. Moreover, the results regarding anxiety are in line with former findings that reported negative relationships of body image facets like SA and levels of anxiety in the general population [33], cancer patients in general [63], and samples of exclusively women with cancer (e.g. [69]). A similar picture emerges for the relationship between cancer-specific distress and female cancer patients’ SA [63, 70]. The role of cancer type will be discussed in detail in the following section on body image in different cancer entities.

#### Factors associated with PA in women

Significant factors associated with more positive PA in women were higher relationship-satisfaction and less cancer-specific distress. This is consistent with the available literature that suggests that a higher overall relationship-satisfaction and increased dyadic coping can help women with cancer feel more accepted by their partners [42]. Moreover, a conjoint process of mutual support was found to be an effective coping strategy in women diagnosed with breast cancer who are in committed relationships [32]. Former studies also showed a negative relationship between the relational aspects of body image and cancer-specific distress as was found in the present study. Interestingly, age did not emerge as a significant factor associated with PA in female cancer patients in the present study even though this could have been expected from the general literature on aging and body image [64, 65] as well as former results of the SIS-D in the general population [33].

### Body image satisfaction in different cancer entities

#### Body image satisfaction in different cancer entities in men

Men did not differ in either their SA nor their PA when the different available cancer entities (prostate/testicular, visceral, and others) were compared. Studies that directly compare men with different cancer entities are scarce but it can be derived from the available literature in individual cancer populations that differences between SA in cancer types might nonetheless exist despite the findings of the present study [60]. Especially body image difficulties in patients with prostate cancer have been studied repeatedly in the past (e.g. [45]) and seem to be especially prevalent when therapies like androgen deprivation therapy are used [71]. There are also indications that men with prostate cancer might indeed have more body image difficulties than men with other types of cancer [10]. Also, men with testicular cancer are reported to experience body image difficulties as well as changes in their relationships and sexuality [72]. The present results should thus be cautiously interpreted as men with prostate/testicular cancer might nevertheless be especially prone to body image difficulties and especially regarding difficulties related to relationships and sexuality. It is nevertheless conceivable that men with a range of other types of cancer that e.g. require the use of a stoma might experience similar decreases in PPA and therefore further comparative research is clearly needed.

#### Body image satisfaction in different cancer entities in women

Women with breast and gynecological cancer and visceral cancer differed regarding their SA in that women with breast and gynecological cancer reported diminished levels of self-acceptance scores compared to women with visceral cancers. Since there are hardly any studies that compared women with different kinds of cancer regarding body image facets, it is particularly difficult to classify the results of the present study in relation to existing research. Nonetheless, it is well known that women with breast cancer often suffer from body image difficulties that are closely related to the cancer treatment and its side effects [11, 20, 44]. As breast and gynecological cancers directly affect primary or secondary sexual characteristics and the SIS-D’s self-acceptance scale includes one’s feeling of sexual desirability, differences to the group of patients whose cancer has less evident impact on their sexuality are comprehensible especially when considering that breast cancer treatment often includes partial or complete mastectomy. In addition, women may experience impairments in their femininity due to cancers affecting the breast or reproductive organs, which also result in reduced SA. In addition, body image is determined not only by physical appearance but also by functioning.

Female patients of the different cancer types did not differ regarding PA in the present study. As there is no literature on differences in the PA facet of body image in women with different cancer entities, a localization of the results of the present study is difficult. However, these results might indicate that female cancer patients’ PA is impaired not because specific parts of the body are affected but because of the more general effects of cancer and its treatment. Here, too, cautious interpretation of the findings and further examination is required to gain deeper understanding on what specifically leads to the body image impairment in different entities of cancer in female patients.

### Body image satisfaction regarding time since diagnosis

Neither men nor women differed regarding SA or PA when those that got their diagnosis within the last year were compared to those that were living with it for longer. Literature on cancer patients’ body image related to the time since diagnosis is rare, thus further research needs to be undertaken to specifically investigate this topic. Nonetheless, a recent review regarding female breast cancer patients of older age by Davis et al. [73] suggests that body image difficulties might persist for a long time when present after therapy and the results of the evaluation study of the *Body Image Scale* indicate that body image difficulties might even grow over time [74]. Another study with female breast cancer patients indicates that different trajectories might exist, depending on different prerequisits at the time of diagnosis [70]. However, all these findings cannot easily be generalized to men, younger women, and patients with a range of different diagnoses and thus need further examination.

### Limitations

Of course, the present study has some limitations, which will be discussed as follows. First of all, the sample size limits the extent to which the results can be generalized beyond the present sample. Additionally, the composition of the sample was not representative regarding the distribution of cancer entities [75, 76]. The sample included a high proportion of female breast cancer patients that have already been subject to the repeated examination of body image in the past. This subsample also varied particularly strongly in the time since diagnosis. Despite our efforts to also recruit in areas where patients with other tumor entities are also treated, women with breast cancer participated significantly more often than patients with other tumor entities. Future studies should target patients with other tumor types even more specifically. Furthermore, grouping different cancers in the visceral cancer group may influence the results, as the individual tumor entities grouped here may have different effects on body image. Future studies should differentially examine body image in different tumor types. Additionally, the different kinds of treatments could not be differentiated in the present study even though these might have been relevant factors for patients’ body image satisfaction. For example, men with prostate cancer might experience different levels of body image dissatisfaction depending on the kind of therapy they receive [77]. Especially androgen-deprivation therapy can lead to psychological and physical issues such as lassitude, loss of energy, anemia, and weight increase it can result in a decrease in overall quality of life and thus body image disturbances [78]. Similar findings in different cancer entities exist [60, 79]. However, the aim of the present study was not to investigate physical functioning, which may be affected by treatment, but subjective satisfaction with one’s body. It may be that other factors besides physical function or changes may play a role in this judgment such as importance of physical appearance, age, etc.. Another limiting aspect is that the physical condition of the participants was not recorded while this has been shown to be a relevant predictor of satisfaction with one’s body (e.g. [16]). Additionally, the role of relationships was not examined in the present study, even though the mere presence of a spouse might positively influence mental health outcomes in cancer patients (e.g. [67]). It must also be noted at this point that all reported findings regarding factors associated with body image satisfaction have to be interpreted carefully as the present study has only a cross-sectional character and thus no inference of the direction of observed correlations can be concluded. It remains thus unclear, whether the examined factors influence body image or vice versa. The classification of less than or more than one year after diagnosis was made for statistical reasons. Future studies should more closely examine the influence of time since diagnosis on body image. In that case, a prospective, longitudinal study would be more appropriate to assess the effects of time since diagnosis. It should also be noted, that differences from the normative values might partly be due to the data being collected in another sample. Lastly, a history of, or current body image or eating disorders, were not assessed. Thus, people with physical health problems, relevant scarring, and body image or eating disorders may have influenced results in uncontrolled ways.

### Strengths

To the authors’ knowledge, this is the first study to examine body image satisfaction in a large population of male and female cancer patients with a wide range of cancer diagnoses while also incorporating the partner-specific relational aspects of body image. In addition, factors associated with SA and PA in men and women were identified. The results thus contribute significantly to the field of psycho-oncology as well as body image research in general. As satisfaction with one’s body is also an important aspect of quality of life, it is important to consider it in psycho-oncological care in addition to other psychological and physical effects of cancer. Furthermore, satisfaction with one’s body also plays a significant role in relationship and sexuality. Moreover, only a few studies have examined body image in men with cancer (e.g.[45, 80]), especially beyond prostate/testicular cancer.

### Clinical implications

The present study adds to the existing literature on body image in cancer patients and suggests that satisfaction with one’s body in cancer patients is generally impaired in a statistically significant way. This indicates that the consideration of the satisfaction with the physical appearance should be an important part of psycho-oncological care regardless of patients’ sex or type of cancer. Possible body image difficulties then need to be addressed by psycho-oncological professionals. Questionnaires such as the SIS could ideally be used to identify body image problems. However, if this is not possible, body image problems should also be asked about in the medical or psycho-oncological interview. As this often also impacts sexual satisfaction, this is an important area to consider in clinical care. Since sexuality is often not addressed, or addressed inadequately, body image issues might also not be raised. It is likely that many patients may also have inhibitions about addressing this topic on their own. Therefore, the recommendation is that the initiative should come from the professional staff. Besides individually tailored interventions on the clinician-patient level, structured and evaluated interventions for individuals as well as groups exist, especially for breast cancer survivors (e.g. [81, 82]).

## Conclusion

The results of the present study suggest that cancer and cancer treatment can potentially lead to a decreased sense of SA and PA not only in women with breast cancer but also in cancer patients across the sexes and with a variety of different cancers entities. Future research is necessary to further examine group differences and predictors of satisfaction with one’s body with longitudinal studies in patients with different kinds of cancers and to investigate the effect of time since diagnosis more deeply. Additionally, research in larger samples that allow differentiation between specific cancer diagnoses and in populations with other chronic medical conditions like transplantation, heart disease, diabetes, and multiple sclerosis should be conducted in the future.

## Supporting information

S1 Dataset This SPSS dataset was used for the multiple imputation in the present study. (SAV)
