# Correction: Body image in patients with different types of cancer

**DOI:** 10.1371/journal.pone.0307673

**Published:** 2024-07-18

**Authors:** Jan Brederecke, Anja Heise, Tanja Zimmermann

In the Body image satisfaction in different cancer entities in women section, the results regarding SA are discussed incorrectly. The correct text in the section is: Women with breast and gynecological cancer and visceral cancer differed regarding their SA in that women with visceral cancers reported diminished levels of self-acceptance scores compared to women with breast and gynecological cancer. Since there are hardly any studies that compared women with different kinds of cancer regarding body image facets, it is particularly difficult to classify the results of the present study in relation to existing research. Nonetheless, it is well known that women with breast cancer often suffer from body image difficulties that are closely related to the cancer treatment and its side effects [20, 44, 73]. As breast and gynecological cancers directly affect primary or secondary sexual characteristics and the SIS-D’s self-acceptance scale includes one’s feeling of sexual desirability, differences to the group of patients whose cancer has less evident impact on their sexuality would be comprehensible especially when considering that breast cancer treatment often includes partial or complete mastectomy. In addition, women may experience impairments in their femininity due to cancers affecting the breast or reproductive organs, which also result in reduced SA. In addition, body image is determined not only by physical appearance but also by functioning. Therefore, assuming that women with breast and gynecologic cancer represent the group most affected by body image altered to the negative, there are at least two possible explanations for the results found in the present sample. One possible explanation arises from the composition of the sample used, in which a large proportion of breast cancer patients came from studies that investigated partnership. Since the presence of a relationship generally has a positive effect on the quality of life of cancer patients [29], this could have resulted in a bias towards more positive SA values. Furthermore, there might be a disproportionate amount of women with especially body image challenging kinds of cancer in the group with visceral cancers as e.g. differences between colorectal cancer patients with and without a stoma have been reported in the past [15]. Of course, all this is speculative and therefore further research regarding SA differences in women with different kinds of cancer are clearly needed.

Female patients of the different cancer types did not differ regarding PA in the present study. As there is no literature on differences in the PA facet of body image in women with different cancer entities, a localization of the results of the present study is difficult. However, these results might indicate that female cancer patients’ PA is impaired not because specific parts of the body are affected but because of the more general effects of cancer and its treatment. Here, too, cautious interpretation of the findings and further examination is required to gain deeper understanding on what specifically leads to the body image impairment in different entities of cancer in female patients.
